# Iterative Learning Impedance for Lower Limb Rehabilitation Robot

**DOI:** 10.1155/2017/6732459

**Published:** 2017-08-01

**Authors:** Chenhui Guo, Shuai Guo, Jiancheng Ji, Fengfeng Xi

**Affiliations:** ^1^School of Mechatronic Engineering and Automation, Shanghai University, Shanghai, China; ^2^Department of Aerospace Engineering, Ryerson University, Toronto, ON, Canada

## Abstract

This paper discusses the problem of squatting training of stroke patients. The main idea is to correct the patient's training trajectory through an iterative learning control (ILC) method. To obtain better rehabilitation effect, a patient will typically be required to practice a reference posture for many times, while most of active training methods can hardly keep the patients training with correct posture. Instead of the conventional ILC strategy, an impedance-based iterative learning method is proposed to regulate the impedance value dynamically and smartly which will help patients correct their posture gradually and perform better. To facilitate impedance-based ILC, we propose two objectives. The first objective is to find the suitable values of impedance based on the ILC scheme. The second objective is to search the moderate learning convergence speed and robustness in the iterative domain. The simulation and experimental results demonstrate that the performance of trajectory tracking will be improved greatly via the proposed algorithm.

## 1. Introduction

Stroke has been the third major cause of permanent disability or death around the world. According to statistics, in China, the incidence of stroke is 1.82%; up to 10.36 million adults, over 40 years of ages, suffer stroke [1]. Retraining movement is a critical part of recovery for the stroke patients, and walking and other standing dynamic balance activities are typically very high on the list of goals for the patients [2]. To make stroke patients regain motion ability and release the burden of physical therapists, many lower limb rehabilitation robots are developed. Among them, Lokomat is a typical representative, which is a kind of external skeletal type lower limb rehabilitation robot, taking hybrid force-position control strategy, is patient-driven, and enables the patient to accomplish free walking movement [3]. The KineAssist is a wheeled mobile robot for gait and balance training, which allows patients to walk freely and provides balance assistance and weight support [4, 5]. Both of them are highly praised and have achieved great results in the rehabilitation field. Gait training is an important section of lower rehabilitation robot, which attracts a lot of researcher's attention. On the opposite, although squatting training is an indispensable section, it is rarely mentioned. Instead of focusing on gait training, we put emphasis on squatting training in this paper, necessary for improving hip and thigh power production [6]. The underlying problem is that it is hard to adapt to different patients and ensure the accuracy of tracking and the safety and comfort of patients and, meanwhile, give necessary assistance to patients.

During rehabilitation training, the robots have to have direct interaction with humans and safety is a critical concern. To ensure the robot's safe interaction with an unknown environment, a form of impedance control was first raised by Hogan in 1985, based on the idea that neither position nor force should be controlled, but rather the dynamic relation between the two [7]. A patient-driven training strategy requires interactive robot-patient control and is mostly achieved by the use of impedance control. Various researches on impedance control for rehabilitation robots have been studied [8–10]. By adjusting the impedance of the robotic rehabilitation devices, the behavior of the robot can be adjusted from very stiff to very compliant. The robot behavior can be made more compliant if the patient is slightly impaired so that the patient can contribute more voluntary effort in the robotic training process. Similarly, the robot behavior can be made stiffer if the patient is unable to achieve the required degree of motion during the robotic training process [11]. Using impedance control is helpful to improve the security and comfort of human-robot interaction, a strategy widely proved.

Iterative learning control (ILC) has become one of the most effective control methodologies in dealing with repeated tracking control problems or periodic disturbance rejection problems [12]. The notion of ILC is that the performance of a system that executes the same task multiple times can be improved by learning from previous execution (trials) [13]. Meanwhile, many ILC algorithms have been proposed to design a purely feedforward action depending solely on the previous control performance [14–17]. The iterative learning controller, a feedforward controller, generates an improved tracking signal over a specific trajectory utilizing past control results to the plant. Since modeling errors are unavoidable, the real ILC system may violate its convergence condition, although the ILC satisfies the condition for a nominal plant model [18, 19]. In practice, a robust control is usually imported, along with the ILC, for system robustness enhancement and better tracking performance [20, 21]. Ahead of the application of ILC, a feedback controller is typically implemented to act as a prestabilizer which will ensure the closed-loop stability and suppress exogenous disturbance by learning from previous iterations, while the iterative learning controller provides improved tracking performance over a specific trajectory utilizing past control results. In the past decade, ILC is introduced into rehabilitation field owing to its learning characteristic. Due to the characteristic of gait motions, Joonbum and Masayoshi proposed a gait rehabilitation strategy that the assistive torque in the current stride is calculated based on the information from the previous strides, inspired by an iterative learning algorithm [22]. RUPERT, an exoskeleton robot used for assisting rehabilitation of arm functions, which has a closed-loop controller combining a PID-based feedback controller and an iterative learning controller based on a feedforward controller, is designed to assist in repetitive therapy tasks related to activities of daily living [23]. Recently, Freeman et al. developed an FES-based upper limb rehabilitation system which can adjust FES signals according to subject's tracking performance through ILC [24]. Joonbum and Masayoshi developed wearable lower limb rehabilitation robots for gait training which will provide smart assistive torque for patients with the help of ILC. Both RUPERT and Freeman et al.'s robots put emphasis on upper limb rehabilitation and combined a feedback controller with ILC to make the system stable.

In this paper, an impedance-based ILC method is proposed and analyzed for the squatting training of stroke patients in the iterative domain and time domain. The method is to correct patient's training trajectory by integrating the ILC scheme with the value of impedance. Specifically, the correction of the training trajectory input for the rehabilitation robot controller is derived by learning the proper impedance value via ILC. By learning the past trajectory tracking information, the proposed ILC method is able to gradually improve the performance of trajectory tracking, and specific training condition of different individuals can be obtained. The convergence and effectiveness of the proposed methods are validated through the results of case studies via simulations and the experiments.

## 2. Lower Limb Rehabilitation Robot System Description

### 2.1. Hardware Description

**Figure 1:**
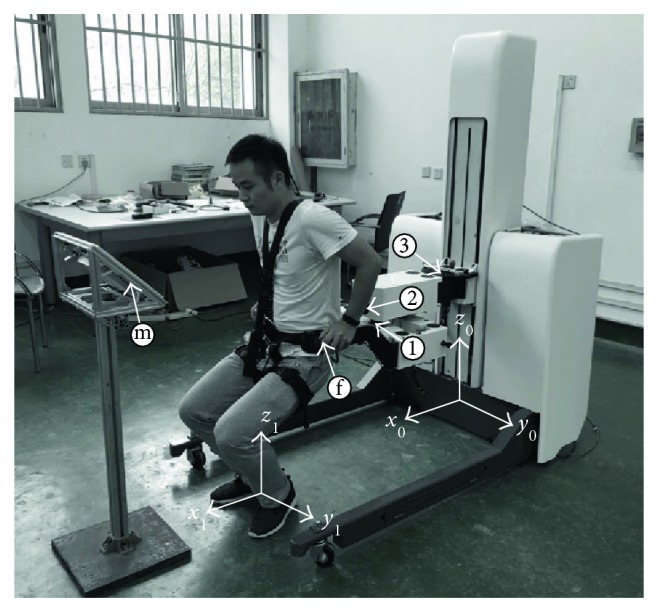
Overview of the rehabilitation system.

As shown in Figure 1, the robot system is mainly composed of omnidirectional mobile chassis (OMC) and body weight support (BWS) system. The OMC consists of two passive wheels and two active wheels, the former ones are installed in the front of the robot which can move in all directions and the latter ones are laid out in the back of the robot. Each active wheel is driven by two independent servo motors, one for driving and the other one is used for steering. Encoders are mounted on each motor shaft to record the rotate angle through which the position of the robot can be calculated. Actually, patients would be divided into different groups taking into account their disability level. The disability level of patients suffering from stroke impairments varies from person to person and also for the same patient during the course of rehabilitation. Patients in high disability level may unable to stand up, not to speak of training, under normal gravity environment. Hence, the BWS system is designed to provide 0–100% body weight support, which can relieve the burden of patient's legs. Furthermore, the BWS system contains 3-DOF (marked as ①, ②, and ③ in Figure 1) to satisfy the demands of free walking and each DOF can be restricted by the locking mechanism. The BWS system is installed on the guide rail, driven by an independent servo motor, which makes it to have one more translational DOF in vertical direction.

At the end of the BWS system, two six-axis force/torque sensors, manufactured by ADI, are installed to record the human-robot interaction force. Forces can be measured of up to 200 N applied in the horizontal plane with a resolution of 0.0122 N. The human subjects will be required to wear a specially designed belt which is strapped on the force sensors tightly. In this case, the end-effector of the robot and human subject is almost overlapped, so we hypothesized that the end-effector of the patient is the same to the robot's. The patient's task is to repeat squatting according to a given trajectory; in addition, the monitor will provide visual feedback of the tracking performance during the training. In the tracking task, the robot's job is to give the patient certain support, to apply assistance during the tracking task, and to move the patient's position when necessary.

### 2.2. Modeling and Linearization

**Figure 2:**
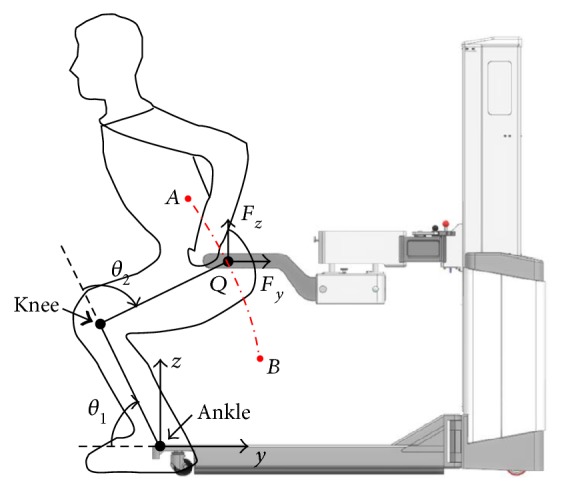
The geometry of the dual human and robotic system.

As shown in Figure 2, it is the geometry of the human and robotic system. During the tracking task, the robot needs to compensate the position of *X*-direction and *Z*-direction when the subject squats. Since the motion in *Y*-direction is unnecessary in this task, the redundant DOF of BWS system are restricted by means of the locking mechanism. Meanwhile, the position of the subject's foot is taken as the origin of the coordinate system, because it is almost fixed on the ground during the training. It is assumed that the subject interacts with the robot by applying a vector of forces and torques at point *Q*, the interaction point, where the forces and torques are measured by two force sensors.

The combined human-robot interaction dynamic model can be described as

(1) $$\begin{array}{c} M\left(q\right)\ddot{q}+V\left(q,\dot{q}\right)\dot{q}+G\left(q\right)=\tau_{d}+\tau_{h}, \end{array}$$

where $q,\dot{q},and\;\ddot{q}$ represent the vectors of robot's position, velocity, and acceleration, respectively. *M* is the system mass matrix, *V* is a Coriolis matrix, and *G* is the gravitational matrix. The matrix *τ*_*h*_ is the vector of interaction torque of the human-robot system. The matrix *τ*_*d*_ represents the vector of impedance, the dynamic relationship between human and robot, used to guide the subject's limbs on reference trajectory.

It is well known that accurate robot motion control requires complex nonlinear controllers [25]. Considering the complexity and uncertainty of modeling, it is difficult to design the controllers. To facilitate the design of controller, we linearized the nonlinear human-robot system and described it as state-space representation [26]. The general state-space representation of a linear system is given as follows:

(2) x˙=Ax+Buy=Cx,

where the term *A* = −*M*^−1^*V*, the term *B* = −*M*^−1^, and the term *C* = diag(1 1). The input term *u*(*t*) represents the torque signal, and the output term *y*(*t*) represents the velocity vector of interaction point *Q*.

### 2.3. Trajectory Planning

**Figure 3:**
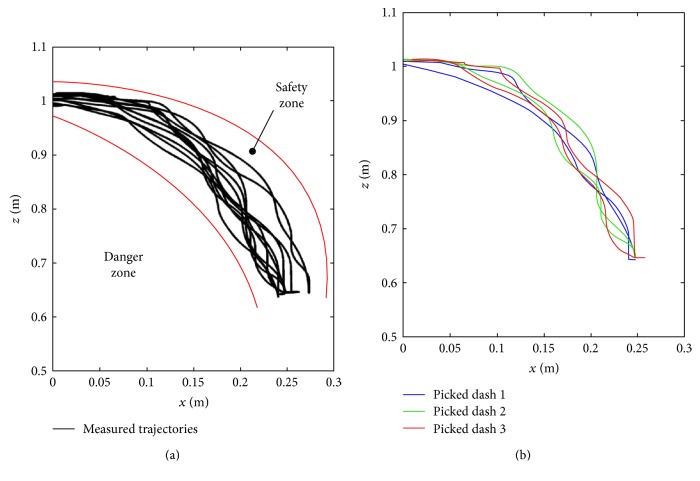
(a) The measured trajectories over 10 times. (b) The squatting trajectories at the first (blue dash), second (green dash), and third (red dash) tracking.

As there is no specific reference trajectory for tracking, an experiment is conducted by a healthy subject to reveal the law of squatting trajectory. In this procedure, the robot is set to following mode to respond to the subject's motion, and encoders' data is stored in real time. As shown in Figure 3, it is a diagram of squatting trajectory generated by recorded position parameters. Although the displayed trajectories shown in Figure 3(a) look confusing, all of the trajectories are inside the safety zone. Different from other trajectory tracking tasks, squatting training is very possible to cause falling down or injury which indicates the given trajectory for human subjects should not cross the safety zone. To have a good knowledge of the variation of each training trajectory, three dashes are picked from Figure 3(a). We can see that the three dashes shown in Figure 3(b) are irregular because the test subject's motion is optional and unrestrained. To make the tracking task easy, the reference trajectory should be smooth and simple. So the idea is that the given trajectory is generated according to the test data; meanwhile, position deviation within a certain range is tolerated which will guarantee the compliance during training.

**Figure 4:**
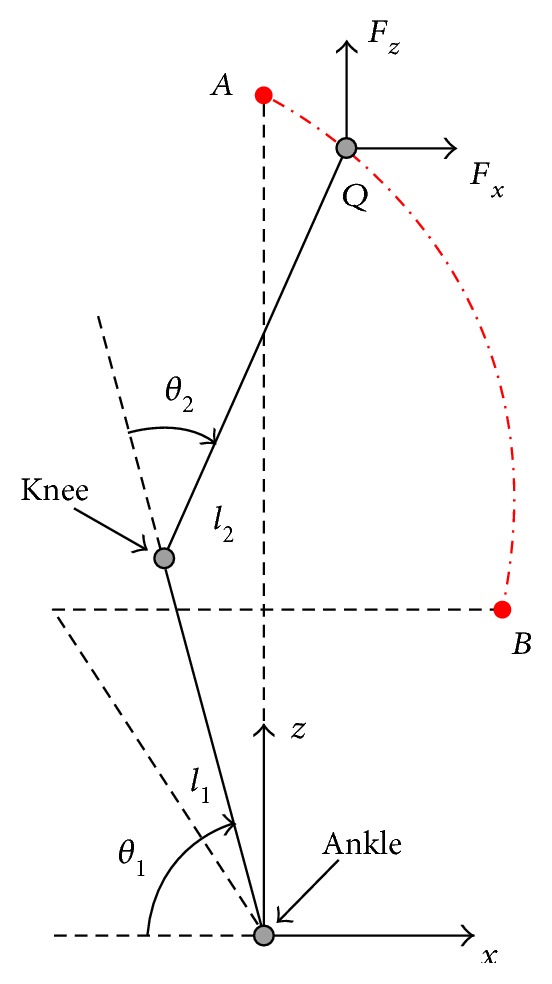
Squatting trajectory in the vertical plane.

As shown in Figure 4, it is a diagram of the human subject's lower limbs. Points A and B represent two extreme positions of training. *L*_1_, the distance from joint ankle to joint knee, is the length of shank, and *L*_2_ is the length of thigh. The human joint angle vector is  *θ* = [*θ*_1_ *θ*_2_]^*T*^, where *θ*_1_ and *θ*_2_ are the joint angles of ankle and knee, respectively. It is found that the training trajectory of subjects varies from person to person while the variation of joint angles is similar. Therefore, the kinematic formulation of squatting trajectory is given as

(3) $$\begin{array}{c} q_{r}=f\left(\theta_{r},l_{r}\right), \end{array}$$

where *q*_*r*_ is a vector that represents the reference training trajectory, *θ*_*r*_ is a vector of the reference joint variables, and *l*_*r*_ is a vector of the kinematic parameters. Given that *l*_*r*_ can be measured in advance, the trajectory of the end-effector is mainly determined by *θ*_*r*_.

The disability level of stroke patients varies from person to person; some patients may be impaired seriously, while the others are impaired slightly. Therefore, taking into account the different disability levels of patients, the time taken, *T*, to travel along the given trajectory, takes a value between 5 and 15 s. Besides, subjects will often be required to track the same trajectory over 20 times. The main idea of this paper is to correct the subjects' posture according to the reference trajectory and make the training process very compliant and comfortable by modifying the term *τ*_*d*_ with iterative learning method.

## 3. Impedance-Based Iterative Learning Control

### 3.1. Impedance Control

A widely used impedance model is given as

(4) $$\begin{array}{c} \tau_{d}=K_{k}\left(q_{r}-q\right)+K_{B}\left(\dot{q_{r}}-\dot{q}\right)+K_{M}\left(\ddot{q_{r}}-\ddot{q}\right). \end{array}$$

The terms *K*_*K*_ = *K*_*K*_*I*, *K*_*B*_ = *K*_*B*_*I*, and *K*_*M*_ = *K*_*M*_*I* are the gain matrices, where *I* is the identity matrix. If we hope the subject moves freely along the trajectory, the gain *K*_*k*_ is often set as 0 and the values of *K*_*B*_ and *K*_*M*_ are assumed to be positive values to create a natural feel. We can let the robot moves the patient's position along predefined trajectories with setting *K*_*k*_ = *K*_*k*_*I* with the scalar *K*_*k*_ > 0. The higher the gain *K*_*k*_ is, the stiffer the system is and vice versa. Therefore, the stiff and compliant characteristic is mainly determined by the gain *K*_*k*_.

As mentioned above, the robot's job is to give subjects help when necessary and guarantee a safe interaction. How the impedance gain is set is a tough task. Adaptive impedance control of the rehabilitation robot is a well-established method to modify the robotic assistance in gait training based on the concept of setting the robotic impedance high (low compliance) if little effort or participation is detected and vice versa [11]. However, high impedance will increase the robotic assistance in order to guide the subject's limbs on reference trajectory which will make the subjects uncomfortable and more likely to fall down. Different from adaptive impedance control method, ILC can reduce position error gradually. The object of this paper is to develop a trajectory corrector that can correct the subject's posture through ILC and improve the subject's tracking performance.

### 3.2. Iterative Learning Impedance

**Figure 5:**
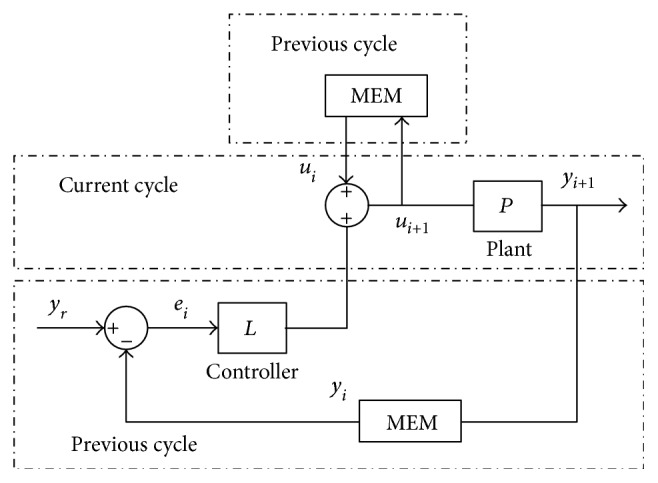
Schematic block of ILC.

Before the discussion of our method, a brief introduction to ILC is presented. As shown in Figure 5, the formulation of ILC is given as follows. Consider the following linear discrete time-invariant system, that is,

(5) $$\begin{array}{c} \begin{array}{c} x\left(t+1\right)=Ax\left(t\right)+Bu\left(t\right)\\ y\left(t\right)=Cx\left(t\right), \end{array} \end{array}$$

where *t* is the time index, *x*(*t*) ∈ *R*^*n*^, *u*(*t*) ∈ *R*^*r*^, and *y*(*t*) ∈ *R*^*r*^ represent the state, control input, and output, respectively. The vectors *A*, *B*, and *C* are matrices with corresponding dimensions. The control target is to find a suitable input *u*_*i*_ (denoted by *u*_*r*_) which produces *y*_*i*_ that precisely follows a reference trajectory *y*_*r*_. The integrator, or *I* term, is rarely used for learning function because ILC has a natural integrator action from one trial to the next [13]. Therefore, the PD-type learning law can be given as

(6) $$\begin{array}{c} u_{i+1}\left(t\right)=u_{i}\left(t\right)+k_{p}e_{i}\left(t\right)+k_{d}{\dot{e}}_{i}\left(t\right), \end{array}$$

where *k*_*p*_ is the proportional gain, *k*_*d*_ is the derivative gain, and *e*_*i*_ = *y*_*r*_ − *y*_*i*_ is the tracking error. From (4) and (6), the impedance learning law can be given as

(7) $$\begin{array}{c} \tau_{d_{i+1}}\left(t\right)=\tau_{d_{i}}\left(t\right)+\Gamma \left({\dot{e}}_{i}\left(t\right)-Re_{i}\left(t\right)\right), \end{array}$$

where Γ and *R* are the learning gain matrix.

### 3.3. Robustness Analysis

The robustness will be discussed as follows. In our case, the human subject will return to the starting point at the end of the tracking cycle so the initial state condition remains the same at each iteration. Then, the output trajectory can be estimated in terms of the desired output trajectory and the initial state error.

**Theorem 1:**

*The initial condition at each iteration is always the same; that is, x*_*i*_(0) = *x*_0_.

If

(8) $$\begin{array}{c} 0<{||I-CB\Gamma ||}_{\infty }\leq \rho <1, \end{array}$$

the update law (6) ensures that

(9) $$\begin{array}{c} \underset{i\to \infty }{\lim}y_{i}\left(t\right)=y_{d}\left(t\right)+e^{Rt}C\left\{x_{0}-x_{d}\left(0\right)\right\}, \end{array}$$

where *τ*_*d*_ is simplified as *y*.

**Proof**

Let *u*_*a*_(*t*) be a control input

(10) $$\begin{array}{c} y_{d}\left(t\right)+e^{Rt}C\left\{x_{0}-x_{d}\left(0\right)\right\}=Ce^{At}x_{0}+C\int_{0}^{t}e^{A\left(t-\theta \right)}Bu_{a}\left(\theta \right)d\theta . \end{array}$$

The problem is equivalent to prove $\underset{i\to \infty }{\lim}u_{i}\left(t\right)=u_{a}\left(t\right)$.

We define

(11) $$\begin{array}{c} \delta u_{i}\left(t\right)=u_{a}\left(t\right)-u_{i}\left(t\right). \end{array}$$

The main idea of the proof is to show that ||δ*u*_*i*+1_(*t*)||_*λ*_ ≤ *ρ*_0_||δ*u*_*i*_(*t*)||_*λ*_, where 0 ≤ *ρ*_0_ < 1.

From (4) and (9), we obtain

(12) $$\begin{array}{c} \delta u_{i+1}\left(t\right)=u_{a}\left(t\right)-u_{i}\left(t\right)-\Gamma \left({\dot{y}}_{d}\left(t\right)-{\dot{y}}_{i}\left(t\right)\right)=\left(I_{r}-CB\Gamma \right)\delta u_{i}\left(t\right)-\Gamma \left(CA-RC\right)\int_{0}^{t}e^{A\left(t-\theta \right)}B\delta u_{i}\left(\theta \right)d\theta . \end{array}$$

Taking the norm ‖·‖_∞_ on both sides of (11), we have

(13) $$\begin{array}{c} {\left\|\delta u_{i+1}\left(t\right)\right\|}_{\infty }\leq {\left\|I_{r}-CB\Gamma \right\|}_{\infty }\cdot {\left\|\delta u_{i}\left(t\right)\right\|}_{\infty }+{\left\|\Gamma \left(CA-RC\right)\right\|}_{\infty }\int_{0}^{t}{\left\|e^{A\left(t-\theta \right)}\right\|}_{\infty }{\left\|B\right\|}_{\infty }{\left\|\delta u_{i}\left(\theta \right)\right\|}_{\infty }d\theta =\rho {\left\|\delta u_{i}\left(t\right)\right\|}_{\infty }+h\int_{0}^{t}e^{a\left(t-\theta \right)}{\left\|\delta u_{i}\left(\theta \right)\right\|}_{\infty }d\theta , \end{array}$$

where *h*≜‖Γ(*CA* − *RC*)‖_∞_ · ‖*B*‖_∞_, and *a*≜‖*A*‖_∞_.

By multiplying both sides of (12) by *e*^−*λ*^*t* and taking the norm ‖·‖_*λ*_,

(14) $$\begin{array}{c} {\left\|\delta u_{i+1}\left(t\right)\right\|}_{\lambda }\leq \underset{0\leq t\leq T}{\max}e^{-\lambda t}{\left\|\delta u_{i+1}\left(t\right)\right\|}_{\infty }\leq {\rho \left\|\delta u_{i}\left(t\right)\right\|}_{\lambda }+h\;\underset{0\leq t\leq T}{\max}\int_{0}^{t}e^{\left(a-\lambda \right)\left(t-\theta \right)}\underset{0\leq t\leq T}{\max}e^{-\lambda \theta }{\left\|\delta u_{i}\left(t\right)\right\|}_{\infty }d\theta =\left(\rho +h\frac{1-e^{\left(a-\lambda \right)T}}{\lambda -a}\right){\left\|\delta u_{i}\left(t\right)\right\|}_{\lambda }. \end{array}$$

Assume that 0 ≤ *ρ* < 1, it is possible to choose *λ* sufficiently large to have

(15) $$\begin{array}{c} \rho_{0}=\rho +h\frac{1-e^{\left(a-\lambda \right)T}}{\lambda -a}<1. \end{array}$$

Thus,

(16) $$\begin{array}{c} \underset{i\to \infty }{\lim}{\left\|\delta u_{i}\left(t\right)\right\|}_{\lambda }=0. \end{array}$$

According to the definition of the norm ‖·‖_*λ*_, these convergence are uniform on *t*  ∈  [0, *T*]. Therefore, $\underset{i\to \infty }{\lim}u_{i}\left(t\right)=u_{a}\left(t\right)$ uniformly on [0, *T*].

From (10), (11), (12), (13), (14), (15), and (16), we have $\underset{i\to \infty }{\lim}y_{i}\left(t\right)=y_{d}\left(t\right)+e^{Rt}C\left\{x_{0}-x_{d}\left(0\right)\right\}$.

Note that, in the proof, the initial state condition of each iteration remains the same. Therefore, the resulting output trajectory can be exactly estimated by the design gain *R* and the initial state error *x*_0_ − *x*_*d*_(0). From (9), if *R* = 0, the converged output trajectory follows the desired trajectory with the offset of the initial error, and if *R* is chosen such that λ(*R*) < 0, the learned control input enables the system to possess an asymptotic tracking capability even in the face of nonzero initial error.

## 4. Simulation and Experiment Results

**Figure 6:**
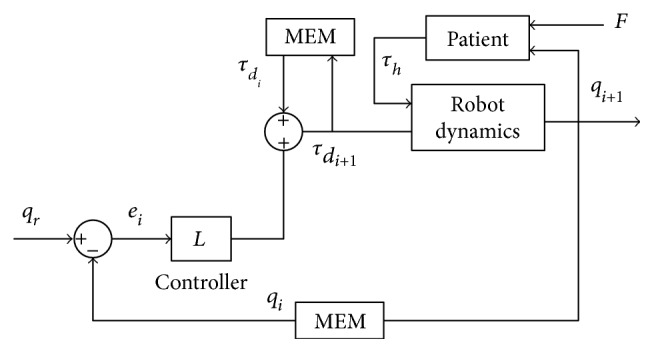
Schematic block of the proposed scheme.

As shown in Figure 6, it is the schematic block of the proposed scheme. The position controller in the overall iterative learning impedance scheme generates the impedance based on the trajectory tracking errors, but does not consider the contribution of human subjects' active force. The ILC block is used to store the input impedance signal in the previous run which will be used in the next run after modified. Thus, the impedance of the robot will, in turn, increase or decrease where the human subject deviates or not. Under the circumstances, the human subjects will move freely in the preliminary stage; then, with the help of the controller, they will get help when they deviate from the predefined trajectory. The more mistakes, the more impedance and vice versa.

The dynamic model is applied in the simulation, and the geometric parameters of human subject are shown in Table 1. The simulation is conducted to validate the proposed algorithm on a MATLAB R2014a with a simulation toolbox. Then, the experiments are complemented on the rehabilitation robot for validating the proposed algorithm.

### 4.1. Simulation Studies

The parameters of the human-robot system in simulation are

(17) $$\begin{array}{c} A=\left[\begin{array}{cc} -0.1695 & 0\\ 0 & -0.1 \end{array}\right],\\ B=\left[\begin{array}{cc} -0.0477 & 0\\ 0 & 3 \end{array}\right]. \end{array}$$

The learning gains of the impedance controller are given as follows:

(18) $$\begin{array}{c} \Gamma =\left[\begin{array}{cc} -10 & 0\\ 0 & 0.5 \end{array}\right]\\ R=\left[\begin{array}{cc} 3 & 0\\ 0 & 2 \end{array}\right], \end{array}$$

where the term Γ and the term *R* satisfy the convergence condition (8).

**Figure 7:**
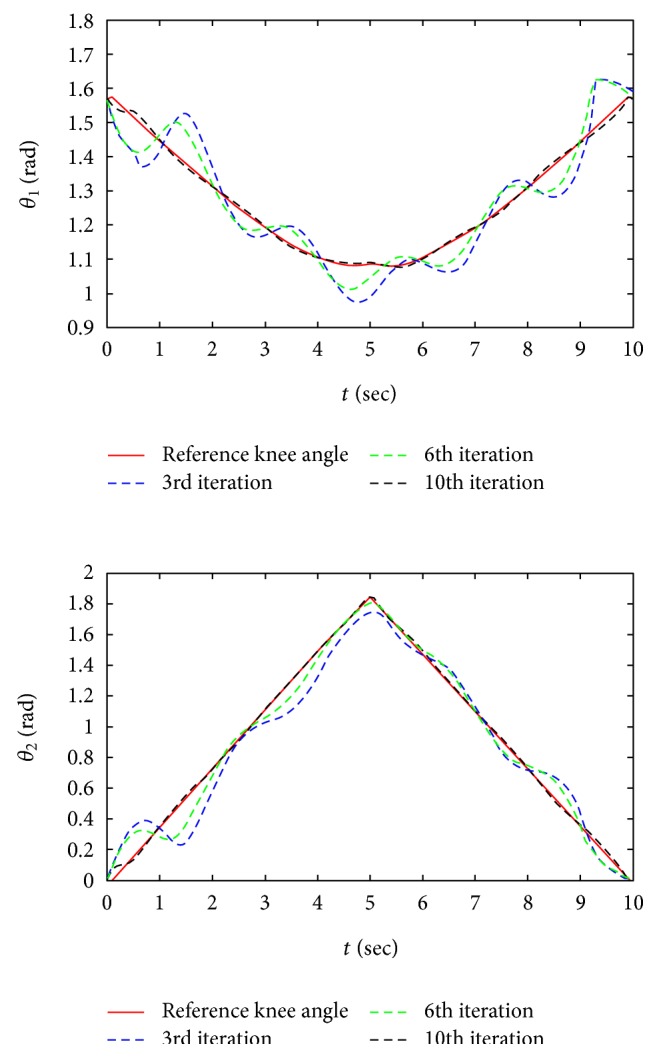
The variation of joint angle in simulation.

**Figure 8:**
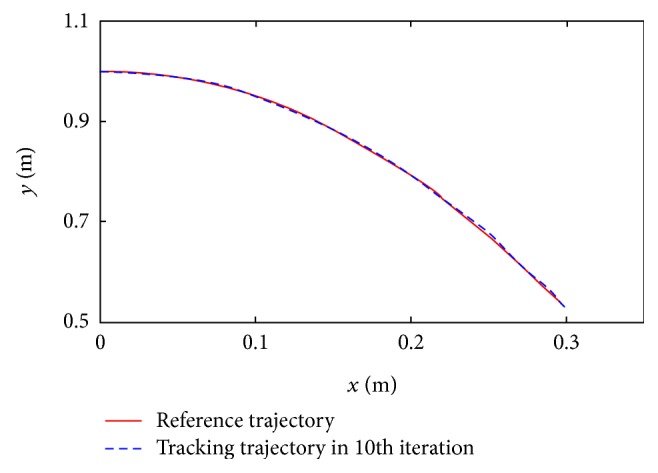
The performance of tracking trajectory in simulation.

In the simulation, the impedance gains are set to make the system compliant and the convergence of the proposed algorithm is verified. Since it is hard to predict the subject's active force, the active force is taken as a repeated disturbance noise 0.5sin3.14*t* in order to verify the effectiveness of the proposed control scheme in an actual work environment. The simulation results are shown in Figures 7 and 8, and the potential problems are discussed in the following.

As shown in Figure 7, it is a diagram of the variation of joint angle calculated by the reverse solution of (1). Although the comparison between desired trajectory and tracking trajectory reflects the quality of the training directly, the tracking performance of joint angle is more important which can provide us more training details especially for doctors. The blue dash, green dash, and black dash represent the variation of joint angle in different iterations. We can learn that the proposed method shows the ability of rejecting a repeating disturbance.

As shown in Figure 8, the tracking performance of reference trajectory and tracking trajectory is compared. We can learn that the tracking errors converge to zero with the increase of iteration.

### 4.2. Experimental Results

As mentioned above, the convergence condition and robustness of the proposed algorithm are proved. Further, we apply the proposed algorithm on the lower limb rehabilitation robot to verify its actual performance. In experiment, the test subject was required to track the reference trajectory 10 times whose geometric parameters are shown in Table 1. Body weight support was not used during the experiments as the test subject was not suffering from stroke or neurologic impairments. Experiments with healthy subject were conducted to evaluate if the iterative learning impedance scheme could modify the robotic assistance based on the past tracking information.

**Figure 9:**
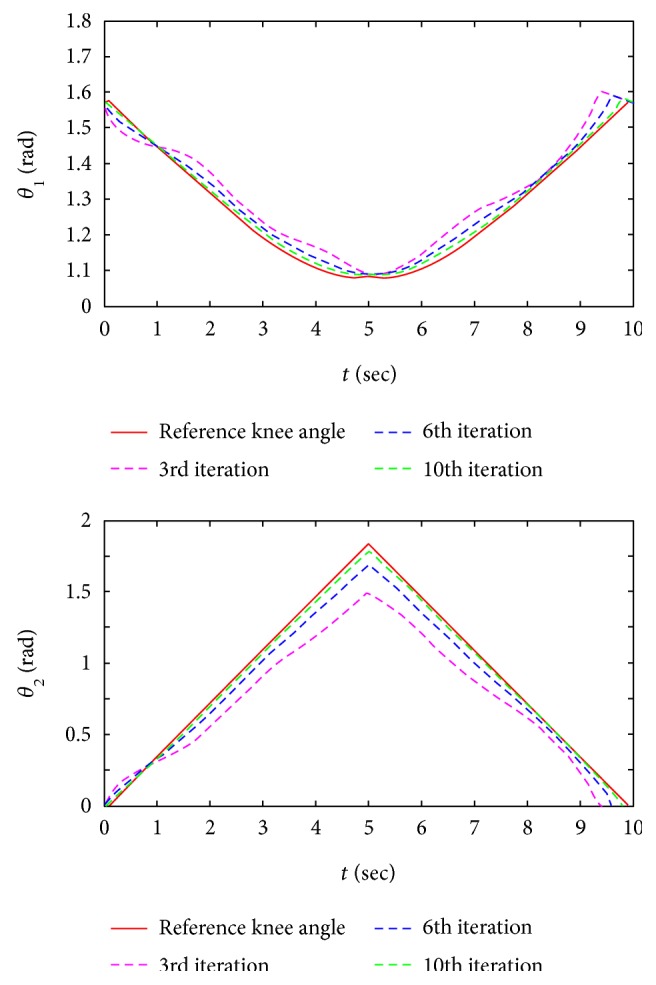
The variation of joint angle in experiment.

As shown in Figure 9, it is a diagram of the variation of joint angle in the experiment. During the first several trials, the test subject's tracking performance of joint angle is not good which indicates that his posture is not correct. With the increase of iteration, the variation of test subject's joint angle approximately approaches to the reference one, which indicates that the subject's training performance is gradually improved under the assistance of the robot.

**Figure 10:**
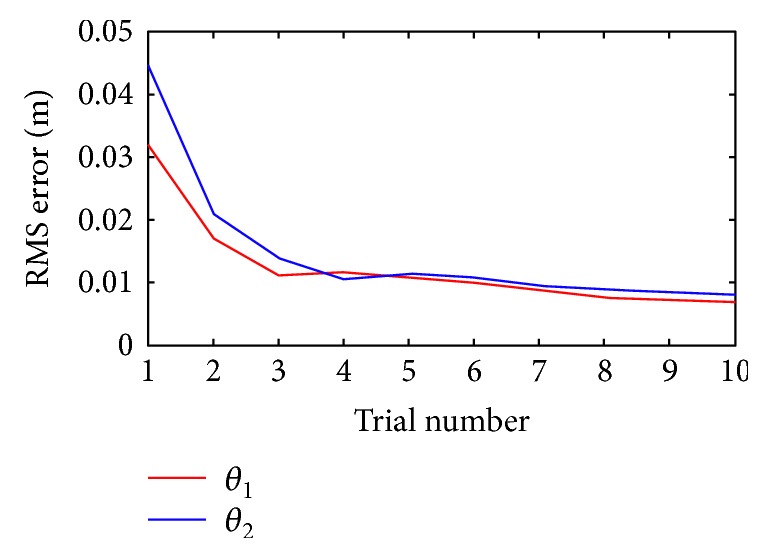
RMS error joint angle.

As shown in Figure 10, it is a diagram of the root mean square (RMS) error corresponding to the proposed method which converges to approximately 8 mm. Considering that the squatting training is a relatively tough task for the subject, error within 15 mm is acceptable. It can be seen that the error reduces rapidly in the first several training cycles which indicates the improvement in tracking accuracy that the PD-type ILC schemes can provide.

**Figure 11:**
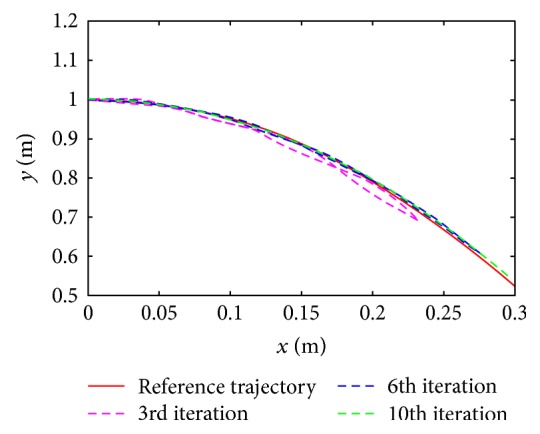
The trajectory tracking performance in experiment.

As shown in Figure 11, the reference trajectory and tracking trajectory of the test subject are compared indicating that the test subject can almost track the reference trajectory accurately in the 10th iteration.

## 5. Conclusion

In the paper, the method of iterative learning impedance has been proposed to support training assistance and correct the patient's posture with the use of the lower limb rehabilitation robot. The convergence condition of the proposed algorithm is given, and the robustness to the parameter variables is analyzed. The simulation and experimental results show that compliancy and assistance have been achieved with the proposed iterative learning method. The most of current rehabilitation robot shows the same problem that it can hardly have both compliancy and robotic assistance. Although many robotic orthosis using impedance control can make the subject track the reference trajectory accurately, many subjects are easy to get into a situation that their limbs are driven by the robot passively. The main idea proposed in this paper introduced a learning impedance method, which can give subjects certain support where they underperform and make them train voluntarily in most training period. The system will enhance the impedance where the subjects deviate by the learning of last operation information. Considering the different geometric parameters of subjects, we proposed a method to generate the training trajectory easily with the absence of any training experiments. Although ILC algorithms have moved beyond these relatively simple structure types and now encompass as wide range of plant models and control law structures, the approach taken here was to apply ILC laws with the simplest structure which could meet the necessary performance requirement. Future works will be extended to the use of different structure types of ILC algorithms such as considering the whole past operation information or taking current iteration structure which will learn the current iteration error by introducing a feedback controller.

## Figures and Tables

**Table 1:** Geometric parameters of human subject.

Gender	Age (years)	Leg length (m)	Thigh length (m)	Height (m)	Body weight (kg)
Male	22	0.42	0.51	1.71	72
